# The Metropolitan Convalescent Institution

**Published:** 1841-10

**Authors:** 


### The Metropolitan Convalescent Institution.

As we have reason to believe that this most excellent institution is much less known to the members of the profession than it ought to be, we beg to call the attention of our readers to the following brief notice of its nature and objects, extracted from the Society's First Report just published. The importance of such an establishment is self-evident; and as we can vouch for the high respect, ability and disinterestedness of the managers of it, we venture to hope that many of our metropolitan readers will be induced to contribute to its funds and also to make known its character to benevolent persons not in the profession. Funds are much wanted to carry its objects into effect.

"The object of the Metropolitan Convalescent Institution is to provide an Asylum in the Country for the temporary accommodation of the convalescents who have been dismissed from our Hospitals and Dispensaries, and for the Sick Poor generally, for whose recovery a short change of air is found to be essential. There are numerous persons who, on leaving the hospital or sick room, are in so destitute and helpless a condition in consequence of the effects of exhaustion and disease, as to be totally unfit to return to their accustomed employments, without their health being more fully established by means of a temporary change of air; whilst there are many others, particularly among the poor, who are labouring under a class of diseases which admit of but little relief from the highest resources of medical skill, and whose only hope of recovery exists in the invigorating influence of country air. Most of these persons are from poverty deprived of the means of obtaining this necessary remedy, and being in consequence of their debility rendered incapable of providing for themselves and families, have no other resource but to become burdens on society, by being the frequent patients of an hospital, or the inmates of a workhouse. It is to provide the means of restoration of health to these persons, many of whom have at one time discharged important duties in life, that the Metropolitan Convalescent Institution has been established.

"The necessity for such an Institution has long been felt by those who have the best opportunity of estimating its importance. A convalescent Institution, indeed, must be regarded as absolutely essential to the completion of our present system of hospitals, and will materially aid in relieving these establishments from the burden of many patients, who having never had the means of acquiring sufficient strength before engaging in their accustomed occupations, become subject to continual relapses, and are frequently obliged to return to the wards of the hospital.

"The Committee have succeeded in obtaining for the Institution very eligible and commodious premises, most agreeably situated in the neighbourhood of the healthy village of Carshalton, in the county of Surrey. The building has garden and other grounds attached, with every necessary convenience, and can accommodate from 50 to 70 persons. Thus, calculating that 50 is the number of patients, and that the average residence of each is one month, there would be upwards of 600 persons annually relieved by means of this Institution. The Committee, however, need scarcely remark, that the extent of relief must altogether depend on the amount of contributions; and they trust that the benevolent will liberally aid them, especially at present, when the furnishing and other necessary expenses will almost exhaust the funds of the Institution.

"Subscriptions will be thankfully received by Messrs. Snow and Co., Bankers, Strand; The Hon. Secretaries, J. C. Pott, Esq., 19, Lincoln's-inn-fields, T. Monro, Esq., 87, Harley-street; Messrs. Mortimer and Haselden, 21, Wigmore-street; and Mr. Nelson, the Collector of the Institution.

---

